# Application of multidimensional gait feature fusion algorithm in gait assessment for patients with knee osteoarthritis

**DOI:** 10.3389/fbioe.2025.1645162

**Published:** 2025-09-19

**Authors:** Yuzhe Tan, Ziyi Wang, Yuhao Wu, Haicheng Wei, Jing Zhao, Yuanyi Jiao, Yu Qin, Yitong Wang

**Affiliations:** ^1^ School of Electrical and Information, North Minzu University, Yinchuan, China; ^2^ School of Medical Technology, North Minzu University, Yinchuan, China; ^3^ School of Information Engineering, Ningxia University, Yinchuan, China

**Keywords:** knee osteoarthritis (KOA), total knee arthroplasty (TKA), hip-knee cyclogram, sample entropy, machine learning, gait assessment, multidimensional gait feature fusion algorithm

## Abstract

**Background:**

To address the limitation in gait assessment for patients with knee osteoarthritis (KOA) and after total knee arthroplasty (TKA), where it is difficult to simultaneously quantify joint dynamic coordination and movement complexity, a multidimensional gait feature fusion algorithm is proposed.

**Methods:**

Spatial motion data were collected from 70 participants (21 healthy controls, 24 KOA patients, and 25 post-TKA patients) using a 3D motion capture system. Hip-knee cyclograms were constructed to extract morphological features (centroid, range of motion, perimeter, and area) for quantifying dynamic coordination, while sample entropy of hip, knee, and ankle joint angles was calculated to quantify movement complexity. Features were categorized into four input types: fused multidimensional features, cyclogram morphological features, sample entropy features, and traditional spatiotemporal parameters. Machine learning models including Random Forest (RF), Support Vector Machine (SVM), Decision Tree (DT), and k- Nearest Neighbors (KNN) were employed for gait classification and assessment.

**Results:**

Multidimensional feature analysis revealed a characteristic pathological compensation pattern of “decreased cyclogram features with increased sample entropy” in the KOA group, while the TKA group demonstrated postoperative improvements in both dimensions. The incorporation of multidimensional features significantly enhanced the performance of all classification models: under multidimensional feature input, RF, SVM, DT, and KNN achieved accuracies of 96.93%, 92.44%, 90.29%, and 88.98%, respectively—all significantly outperforming models using single‐dimensional features.

**Conclusion:**

The multidimensional gait feature fusion algorithm effectively overcomes the limitation of assessing either coordination or complexity in isolation, providing an interpretable quantitative tool for analyzing KOA pathological mechanisms and dynamically monitoring post-TKA rehabilitation.

## 1 Introduction

Knee osteoarthritis (KOA) is one of the leading causes of disability among the elderly (Ro et al., 2019), Its pathological characteristics include not only joint pain and limited mobility but also significant disruption of lower-limb joint dynamic coordination, leading to distinctive gait abnormalities (Liao et al., 2025). Total knee arthroplasty (TKA), an effective treatment for end-stage KOA, can substantially alleviate pain and restore joint range of motion (RoM) (Konnyu et al., 2023), However, postoperative gait coordination and movement complexity in TKA patients remain significantly different from those of healthy individuals. Studies indicate that coordinated movement of the hip, knee, and ankle joints during the gait cycle is crucial for stable walking, and their angular dynamics systematically reflect lower-limb coordination (Zhang et al., 2021). Current clinical assessments primarily rely on imaging examinations such as X-rays (Moura et al., 2025), yet these methods are subject to interpretation subjectivity and struggle to reveal functional changes during disease progression, highlighting an urgent need to establish objective and multidimensional gait assessment approaches.

In the field of quantitative gait analysis, traditional spatiotemporal parameters can effectively distinguish pathological gait but are limited by their discrete, single-point measurement nature, failing to comprehensively capture the dynamic coordination mechanisms of multi-joint synergistic movements (Pau et al., 2022a). Existing research approaches mainly fall into two categories: (1) phase-based coordination analysis using cyclograms and (2) nonlinear dynamic assessments of movement complexity. Cyclograms, which visually characterize inter-joint coordination patterns, have been widely adopted since Grieve first proposed the theory (Xu et al., 2023). Subsequent studies expanded their applications: Xu et al. (2025) constructed hip-knee coordination metrics using perimeter and area; Park et al. (2021) quantified gait deviations in KOA patients via cyclogram coefficient of variation; Zelik’s team (Zelik et al., 2015) advanced the understanding of gait biomechanics through 6-degree-of-freedom multi-joint analysis. For movement complexity, Sample Entropy (SE), due to its sensitivity to nonlinear characteristics of time series, was demonstrated by Shan et al. (2025) to effectively quantify differences in trunk segment movement complexity between individuals with spinal cord injuries and healthy controls under various perturbation directions, revealing flexibility and adaptive changes in motor control, thereby successfully identifying alterations in movement complexity during challenging seated perturbations in individuals with spinal cord injuries. However, existing studies rarely combine it with cyclogram morphological parameters for joint analysis.

With the penetration of machine learning technologies, preliminary progress has been made in gait feature fusion analysis (Lee et al., 2021). Li et al. (2019) achieved 94% accuracy by combining dynamic time warping (DTW) with sample entropy; Mekni et al. (2025) utilized linear discriminant analysis (LDA) combined with principal component analysis (PCA) to achieve 97.14% accuracy in gait cycle phase classification, demonstrating the effectiveness of combining single algorithms with data preprocessing. Chen et al. (2022) achieved 94.9% classification accuracy using support vector machines based on lower limb joint angle features, confirming the validity of single kinematic parameters. However, existing methods predominantly focus on single-modal features and lack systematic integration of multidimensional information such as cyclogram morphology and joint movement complexity, limiting the analytical dimensions for gait abnormalities in KOA/TKA populations.

To address the aforementioned limitations in gait assessment methods, this study proposes a multidimensional gait feature fusion algorithm. By integrating joint range of motion (RoM), centroid (CoM), perimeter, and area derived from cyclograms with sample entropy features of joint angles, a multidimensional evaluation framework encompassing “dynamic coordination–movement complexity” was constructed. Joint angle data were normalized using cubic spline interpolation and combined with hip-knee cyclograms to achieve full-chain analysis of three-joint synergistic mechanisms, thereby addressing the existing research gap in accounting for ankle joint contributions. Wrapper-based feature selection was employed to optimize inputs for Random Forest (RF), Support Vector Machine (SVM), Decision Tree (DT), and k-Nearest Neighbors (KNN) algorithms, enabling comparative validation of the synergistic enhancement effects of multidimensional features for KOA and TKA rehabilitation assessment. This approach provides an interpretable quantitative tool for elucidating KOA pathology and evaluating post-TKA rehabilitation.

## 2 Materials and methods

### 2.1 Participants

This study was approved by the Ethics Committee of Ningxia Hui Autonomous Region People’s Hospital (Approval No.: 2024-KJCG-001). A total of 70 participants were recruited, including 21 healthy volunteers (Healthy group), 24 knee osteoarthritis patients (KOA group) treated at the hospital, and 25 total knee arthroplasty patients (TKA group) who underwent surgery at the hospital, as shown in Table 1. Among them, five patients in the TKA group received bilateral knee arthroplasty, while the remaining 20 underwent unilateral knee arthroplasty (11 left and 9 right). All KOA patients met the diagnostic criteria of the 2021 edition of the “Chinese Medical Association Orthopedics Branch Osteoarthritis Diagnosis and Treatment Guidelines” with Kellgren-Lawrence grades of 3–4. A total of 14 cases had bilateral involvement, while the remaining 10 cases were unilateral, with seven affecting the left side and three affecting the right side. TKA patients were all end-stage knee disease patients who failed conservative treatment, with postoperative time within 3 months (Ma et al., 2022). All patients were excluded for systemic diseases such as diabetes and rheumatoid arthritis, and signed informed consent forms. This study was strictly conducted in accordance with the ethical guidelines of the Declaration of Helsinki.

**TABLE 1:** Participant anthropometry.

Group	n	Age	Height (cm)	Weight (kg)	BMI	K-L (3–4)	Postoperative time
Healthy	21	56.6 ± 13.8	162.5 ± 8.5	66.7 ± 11.0	25.1 ± 2.9	-	-
KOA	24	58.9 ± 11.4	160.2 ± 8.8	71.6 ± 7.8	26.0 ± 0.3	14/10	-
TKA	25	65.2 ± 10.1	161.2 ± 9.0	71.4 ± 15.2	27.2 ± 3.5	-	2.5 ± 0.8

### 2.2 Equipment and measurement method

**FIGURE 1:**
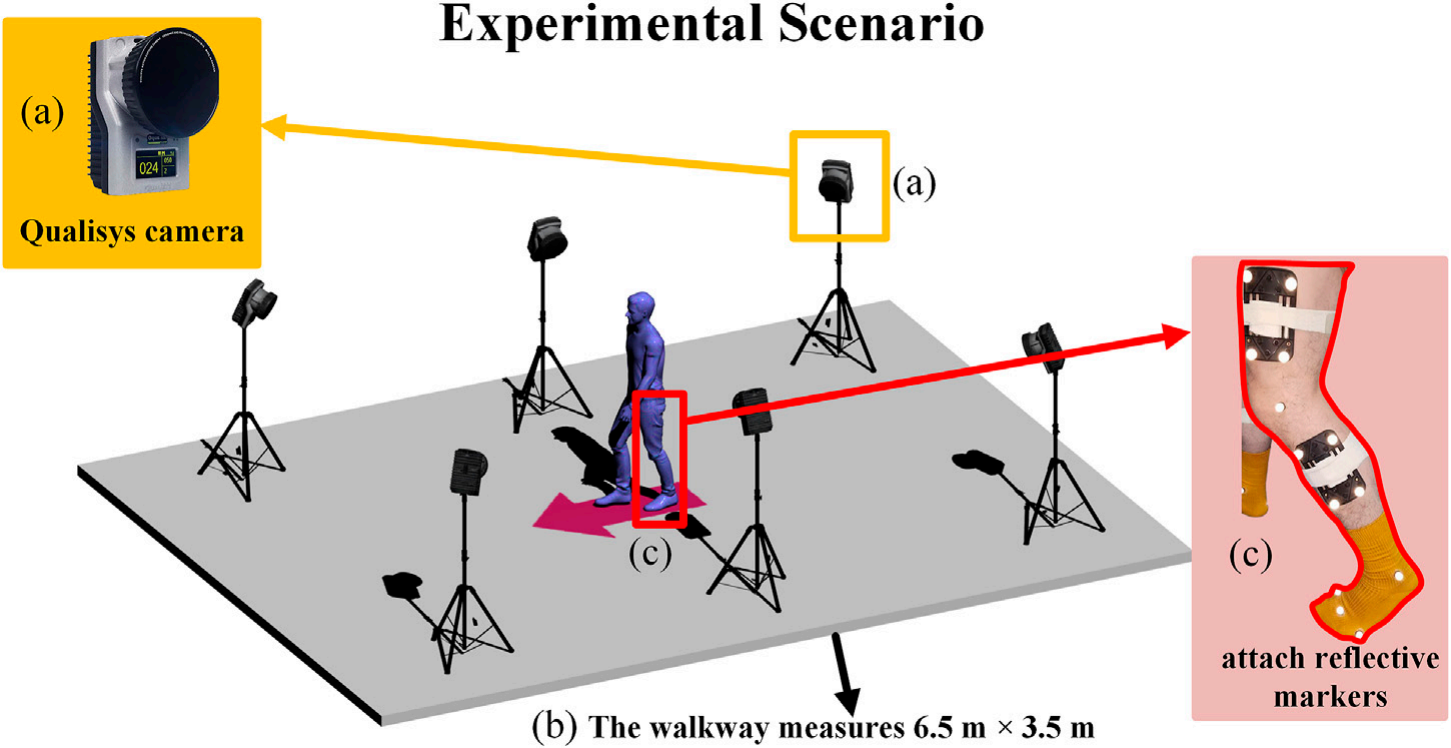
Experimental setup depicting a walkway **(b)** measuring six and a half meters by three and a half meters with a blue humanoid figure surrounded by cameras on tripods. The inset **(a)** shows a Qualisys camera, and inset **(c)** highlights a leg with attached reflective markers.

The experimental setup is shown in Figure 1. This study employed a Qualisys motion capture system (Model: Oqus 700) with six high-speed cameras, as illustrated in Figure 1a. The cameras were arranged in a circumferential layout surrounding a 6.5 m × 3.5 m testing area, as shown in Figure 1b. Three-dimensional spatial calibration was first performed using a calibration wand to ensure capture accuracy. Reflective markers were attached to key anatomical landmarks on subjects, including the pelvis (iliac crest, greater trochanter) and lower limbs (femoral epicondyles, medial and lateral malleoli), as demonstrated in Figure 1c. Prior to formal testing, subjects underwent 5–10 min of adaptive walking training to familiarize themselves with the markers and eliminate potential gait disturbances caused by nervousness. During actual testing, redundant markers were removed, retaining only essential dynamic tracking points while subjects walked naturally on the walkway. The system randomly selected 3-5 complete gait cycles for data collection to ensure natural and representative data. During kinematic reconstruction using TrackManager software, the system’s built-in real-time trajectory optimization algorithm automatically corrected marker drift caused by soft tissue artifacts. By digitally reconstructing lower body anatomical structures, angular changes in the hip, knee, and ankle joints were calculated. The system ultimately exported spatiotemporal parameters and kinematic characteristic data of gait.

### 2.3 Multidimensional gait feature fusion algorithm

**FIGURE 2:**
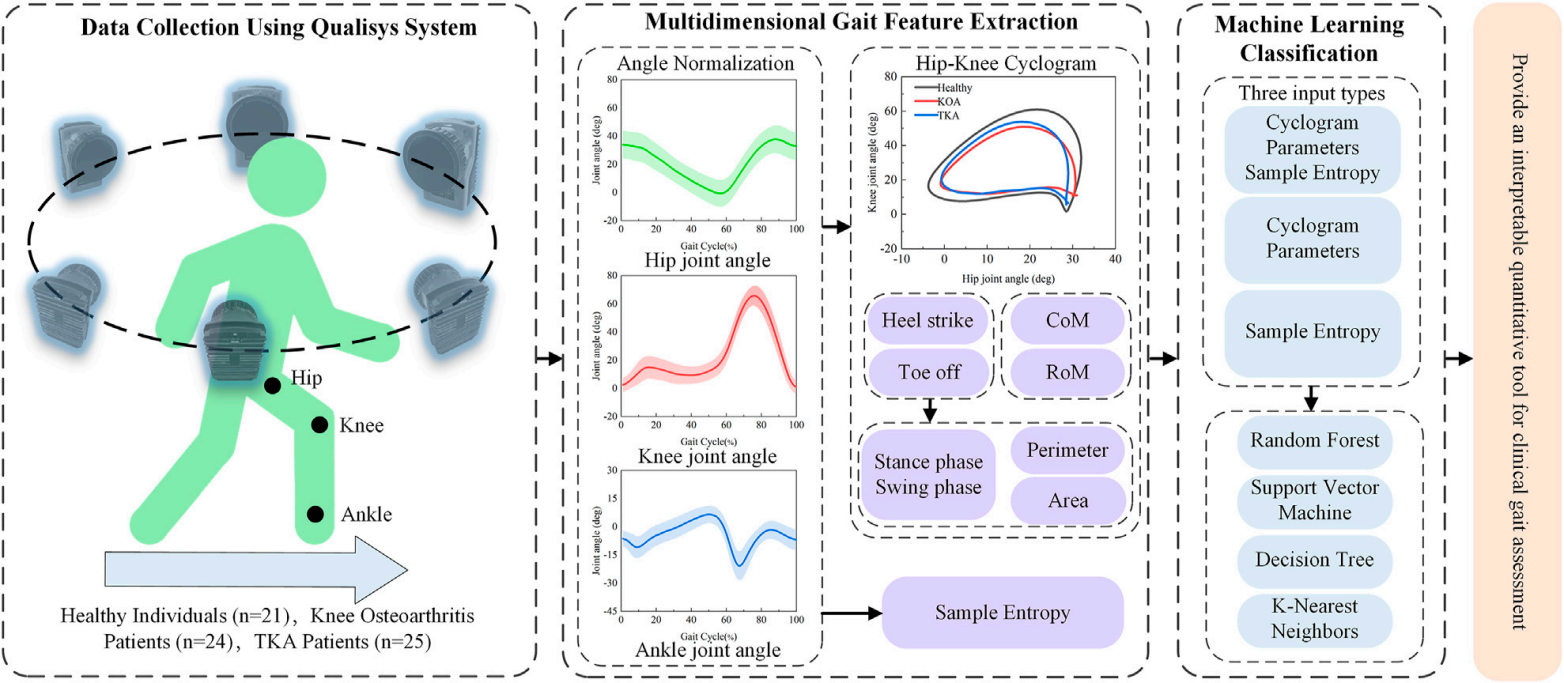
Multidimensional gait feature fusion algorithm.

The multidimensional gait feature fusion algorithm is illustrated in Figure 2. First, gait data from the Healthy group, KOA group, and TKA group were obtained using the Qualisys 3D motion capture system, with joint angle signals normalized to standard gait cycles through cubic spline interpolation. Second, precise gait cycle segmentation was achieved based on extreme value detection of ankle joint angles, identifying heel strike and toe-off events, which were then used to construct hip-knee cyclograms. Morphological features including range of motion (RoM), center of mass (CoM), perimeter, and area were extracted from these cyclograms. Simultaneously, sample entropy of hip, knee, and ankle joint angle sequences was calculated to quantify movement complexity. Finally, four machine learning models (RF, SVM, etc.) combined with wrapper-based feature selection methods were employed to evaluate the classification performance of multidimensional gait features, establishing a comprehensive evaluation system that integrates dynamic coordination and movement complexity. This system provides an interpretable quantitative tool for clinical gait assessment.

#### 2.3.1 Multidimensional gait feature extraction based on cyclogram and sample entropy

After acquiring gait data, considering individual differences in subjects’ height, weight, and gait characteristics that lead to inconsistent numbers of collected data points, the cubic spline interpolation method was employed. Through constructing piecewise cubic polynomials in each subinterval $\left[x_{i},x_{i+1}\right]$ , Equation 1 was obtained:

$$s\left(x\right)=a_{i}+b_{i}\left(x-x_{i}\right)+c_{i}{\left(x-x_{i}\right)}^{2}+d_{i}{\left(x-x_{i}\right)}^{3}$$ (1)

where, $x_{i}<x<x_{i+1}$ and $a_{i}$ are the function values $x_{i}$ at node, $b_{i}$ is the first derivative value, $c_{i}$ and $d_{i}$ are coefficients related to the second and third derivatives, respectively. By ensuring continuity of function values and their first and second derivatives, smooth resampling of non-uniformly sampled data was achieved.

To resolve the difficulty in accurately determining gait events from hip-knee joint angle curves, ankle joint angle changes were utilized as the primary marker: the heel strike moment corresponds to the initiation point of ankle joint angle change, which also represents the starting point of the stance phase, while the toe-off moment corresponds to the maximum plantar flexion angle of the ankle joint, namely, the starting point of the swing phase. The stance phase constitutes approximately 60% of the entire gait cycle, after which the ankle joint rapidly returns to dorsiflexion entering the swing phase that accounts for about 40%. On this basis, sagittal plane hip-knee cyclograms were constructed by plotting the angle change curves of the hip joint (X-axis) versus the knee joint (Y-axis) in a clockwise direction (Saegner et al., 2024).

Since the cyclogram consists of a series of continuous data points, its perimeter is obtained by calculating the linear distance between every two adjacent data points and accumulating them, where the last term is calculated by the distance between the first and last data points to ensure the cyclogram is closed, but this alone cannot guarantee the symmetry of the cyclogram. Specifically, as shown Equation 2:

$$P=\sum_{i=1}^{n-1}\sqrt{{\left(\theta_{hi+1}-\theta_{hi}\right)}^{2}+{\left(\theta_{ki+1}-\theta_{ki}\right)}^{2}}+\sqrt{{\left(\theta_{h1}-\theta_{hn}\right)}^{2}+{\left(\theta_{k1}-\theta_{kn}\right)}^{2}}$$ (2)

In the equation, n is the number of data points, $\theta_{hi}$ and $\theta_{ki}$ are the hip joint angle and knee joint angle of the i-th data point respectively; $\theta_{hi+1}$ and $\theta_{ki+1}$ are the angles of the (i+1)-th data point; $\theta_{h1}$ , $\theta_{k1}$ are the angles of the first point; $\theta_{hn}$ , $\theta_{kn}$ are the angles of the last point; the last term calculates the cross product of vectors from the last data point back to the first data point. The inclusion of this term ensures the sequence of data points forms a closed loop, thereby creating a closed shape, though this alone does not guarantee symmetry of the cyclogram. The cyclogram area A can be calculated using Equation 3:

$$A=\frac{1}{2}\left|\sum_{i=1}^{n-1}\left(\theta_{\text{hi}}\theta_{ki+1}-\theta_{ki}\theta_{hi+1}\right)+\left(\theta_{hn}\theta_{k1}-\theta_{hn}\theta_{h1}\right)\right|$$ (3)

In Equations 4, 5, for each gait cycle i , the mean hip joint angle $cycleCoMx_{i}$ and mean knee joint angle $cycleCoMy_{i}$ are calculated:

$$\text{cy}cleCoMx_{i}=\frac{1}{n}\sum_{j=1}^{n}hipAngles_{j,i}$$ (4)

$$\text{cy}cleCoMy_{i}=\frac{1}{n}\sum_{j=1}^{n}KneeAngles_{j,i}$$ (5)

where n is the number of angle values in each gait cycle, and j represents the jth angle value in the current gait cycle n. In Equations 6, 7, hip joint RoM and knee joint RoM are calculated by subtracting the minimum angle from the maximum angle for each joint, respectively:

$$hipRoM=\max\left(hipAngles\right)-\min\left(hipAngles\right)$$ (6)

$$kneeRoM=\max\left(kneeAngles\right)-\min\left(kneeAngles\right)$$ (7)

Sample entropy is a method for quantifying time series complexity and regularity (Shan et al., 2025), useful for assessing joint movement variability. By calculating sample entropy of hip, knee, and ankle joint angle changes, a more comprehensive understanding of these joints’ coordination and stability can be obtained. The specific implementation is shown in Equation 8, where m is the embedding dimension (vector length), r is the similarity tolerance threshold, N is the total length of the time series, $\phi^{m}\left(r\right)$ represents the average similarity probability between all vectors of length m under given tolerance r, and the sample entropy is $SampEn\left(m,r,N\right)$ .

$$SampEn\left(m,r,N\right)=-\ln\left(\frac{\phi^{m+1}\left(r\right)}{\phi^{m}\left(r\right)}\right)$$ (8)

An embedding dimension of m = 2 effectively captures short-term dynamic patterns in joint angle time series while avoiding template sparsity issues caused by higher dimensions. The similarity tolerance of r = 0.1, adapted to the dynamic range of the data, balances sensitivity and specificity, preventing both noise interference from small tolerances and pattern generalization from large tolerances. The parameter selection followed standard criteria for biomechanical signal analysis and was validated through parameter sensitivity analysis, demonstrating stable ranking of joint entropy values within reasonable fluctuation ranges.

When performing statistical analysis on sample entropy and cyclogram features, the Shapiro-Wilk test was first used to assess data normality. Since the results did not follow a normal distribution, the Kruskal–Wallis test was employed for comparisons of continuous variables between groups, while Fisher’s exact test was used for categorical variables. For variables showing significant differences, pairwise comparisons were further conducted using the Mann-Whitney U test with Bonferroni correction (adjusted p < 0.017). To control for the effects of confounding factors such as age, sex, and BMI, as well as population heterogeneity arising from the affected side in KOA and the surgical side in TKA, analysis of covariance (ANCOVA) was subsequently applied to evaluate intergroup differences after adjusting for the aforementioned variables. For significant main effects identified in ANCOVA, Tukey’s HSD *post hoc* test was performed. Unilateral and bilateral involvement in the KOA group were treated as independent categories; similarly, for the surgical side in the TKA group, unilateral and bilateral surgeries were distinguished, and these detailed side-specific classifications were included as categorical covariates in the ANCOVA model to statistically adjust for their effects on gait features. All analyses were performed using SPSS 27 and the Python statsmodels library.

#### 2.3.2 Machine learning modeling

**FIGURE 3:**
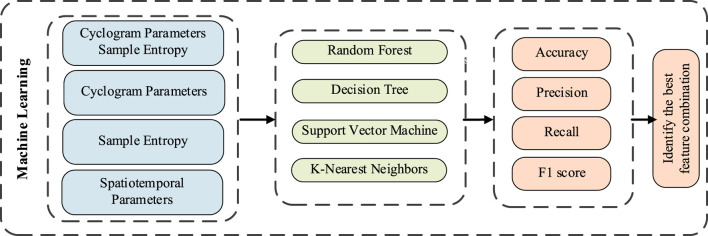
Machine learning evaluation method.

To systematically evaluate the classification efficacy of different gait feature combinations, four machine learning models—RF, SVM, DT, and KNN were constructed (Dibbern et al., 2025), with the complete evaluation process illustrated in Figure 3. To comprehensively validate the superiority of the multidimensional feature fusion algorithm, the input features for the models were categorized into four types for comparative analysis: the first category combined multidimensional features integrating cyclogram morphology and sample entropy, the second category included only cyclogram morphological features, the third category consisted solely of sample entropy features, and the fourth category comprised traditional spatiotemporal parameters.

Prior to modeling, the dynamic gait data of each subject were transformed into a static feature vector, collectively forming an input matrix with subjects as samples and corresponding gait metrics as features. The preprocessing phase followed a systematic pipeline: first, missing values were handled using median imputation, and Z-score standardization was applied to eliminate scale differences. Then, dimensionality reduction was performed by selecting the top 10 features with the highest F-statistics based on ANOVA (Nam Nguyen et al., 2020). A nested stratified cross-validation framework was employed to ensure evaluation robustness: the outer layer used 5-fold stratified cross-validation with 80% training set and 20% test set in each fold, while the inner layer utilized 3-fold cross-validation for hyperparameter optimization. The model parameter configurations were systematically optimized as shown in Table 2, with hyperparameter tuning applied to identify the optimal parameter combinations and maximize model generalization performance. RF employed random search to optimize the number of trees and maximum depth; SVM optimized the penalty coefficient C and kernel function through cross-validation; DT applied pruning strategies to adjust maximum depth and splitting criterion; KNN optimized the number of neighbors and weighting function based on distance weighting (Simon et al., 2023). To address the mild class imbalance among the Healthy, KOA, and TKA groups, a dual strategy was implemented: 1. Stratified sampling ensured that each cross-validation fold maintained the original distribution; 2. A class weighting mechanism adjusted decision boundaries, with SVM employing customized penalty weights. Final performance evaluation used weighted precision, recall, and F1-score to eliminate class bias and ensure robust results.

**TABLE 2:** Configuration parameters of machine learning models.

Model	Key parameters	Tuning range	Optimal parameters	Optimization method
RF	Number of trees/Max depth	50–100/3–5	80/4	Grid search
SVM	C-value/Kernel function	0.1–10/rbf	5/rbf	Cross-validation
DT	Max depth/Splitting criterion	3–5/gini	3/gini	Pruning optimization
KNN	Number of neighbors/Weight	3–7/distance	5/distance	Distance weighting

## 3 Results

### 3.1 Dynamic coordination differences based on cyclogram analysis

**FIGURE 4:**
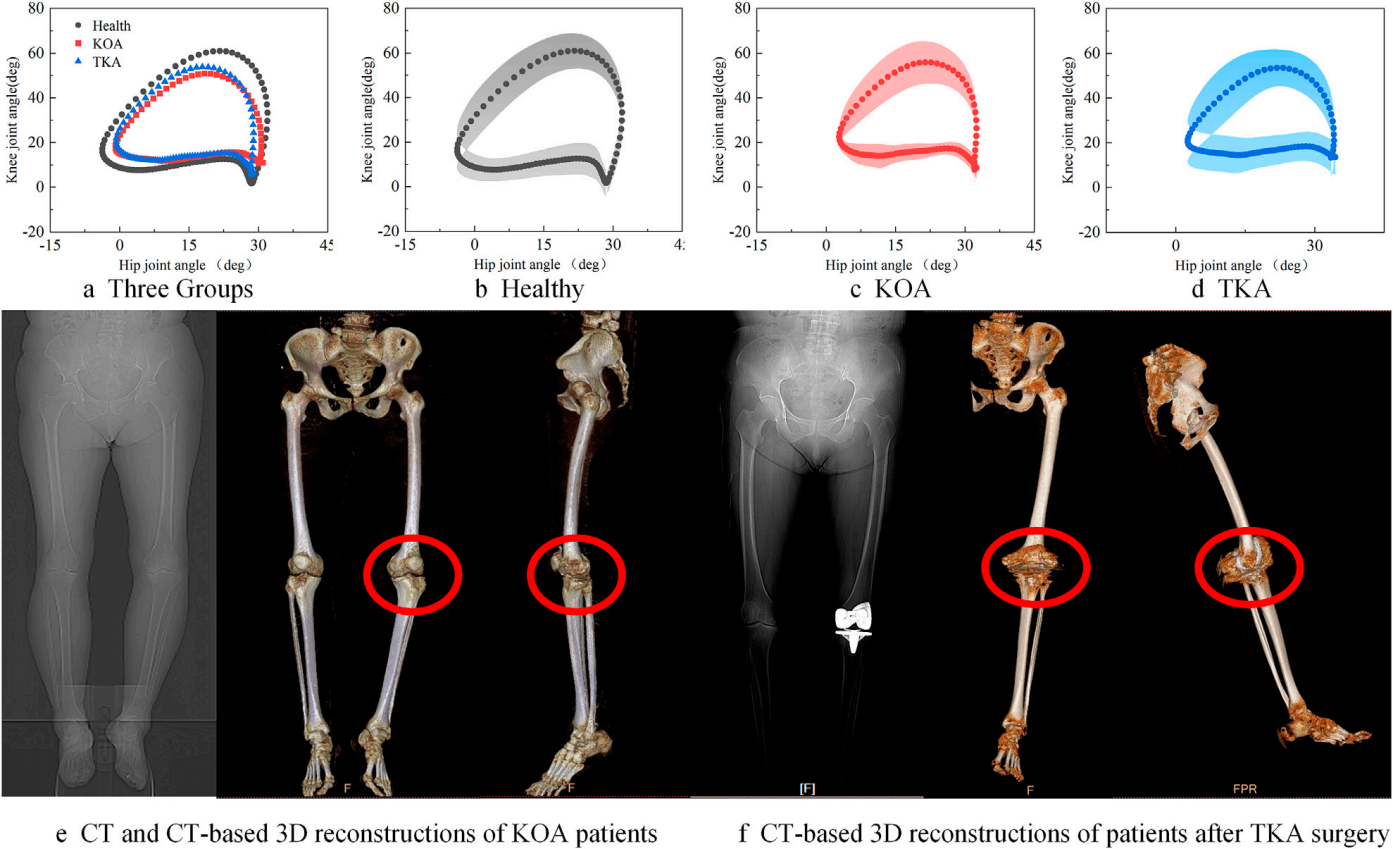
The average hip-knee angle cyclograms for the Healthy, KOA, and TKA groups **(a)**, Average hip–knee cyclogram of the Healthy group **(b)**, Average hip–knee cyclogram of the KOA group **(c)**, Average hip–knee cyclogram of the TKA group **(d)**, CT and 3D-reconstruction images of a KOA patient **(e)**, CT and 3D reconstruction images of a post-TKA patient **(f)**.

Hip-knee cyclogram analysis revealed significant movement coordination differences among the three groups, as shown in Figure 4. The average hip-knee angle cyclograms for the Healthy, KOA, and TKA groups are presented in (a); the Healthy group (b) exhibited typical elliptical trajectories, while the KOA group (c) showed trajectory compression and irregular morphology. Although the cyclograms of the TKA group (d) remained smaller than those of the Healthy group, their trajectory errors were significantly reduced compared to the KOA group, with a slight increase in area, suggesting partial restoration of joint movement coordination post-operation. This visual difference was further validated in the CT and 3D reconstruction images of KOA patients (e), which displayed evident knee varus deformity and joint space narrowing. In contrast, the CT and 3D reconstruction images of post-TKA patients (f) demonstrated proper alignment of the prosthetic joints and significant improvement in lower limb mechanical alignment.

Table 3 quantifies the differences in hip-knee cyclogram characteristics. The knee joint RoM of the Healthy group was 61.45° ± 1.63°, significantly higher than that of the KOA group 50.59° ± 2.61° and the TKA group 44.63° ± 2.46°, indicating significant limitations in joint range of motion in both the KOA pathological state and the early postoperative period. The swing phase perimeter and area of the KOA group decreased by 38.7% and 36.3%, respectively, compared to the Healthy group, with significantly greater reductions than those observed in the stance phase perimeter and area, suggesting that impaired active movement ability is more severe than the decline in stability during the weight-bearing phase. The total perimeter and swing phase perimeter of the TKA group remained significantly lower than those of the Healthy group and showed no statistical difference compared to the KOA group, indicating that joint coordination had not fully recovered postoperatively. The stance phase perimeter and area of the TKA group were significantly reduced by 17.5% and 27.7%, respectively, compared to the Healthy group, demonstrating limited compensatory muscle co-contraction. The total area of the KOA group was significantly reduced by 32.8% compared to the Healthy group, confirming that KOA causes impairment in spatiotemporal coordination ability across multiple lower limb joints. Although the total area of the TKA group showed a slight increase compared to the KOA group, it remained significantly reduced by 30.9% compared to the Healthy group, indicating that the surgery only partially improved compensatory patterns.

**TABLE 3:** Cyclogram characteristics.

Features	Healthy	KOA	TKA	P-value	Multiple comparison		
					HvsK	HvsT	KvsT
RoM(deg)							
Hip	37.30 ± 1.05	33.55 ± 1.25	33.77 ± 1.34	0.067	-	-	-
Knee	61.45 ± 1.63	50.59 ± 2.61	44.63 ± 2.46	**<0.001** ^a^	**0.002** ^a^	**<0.001** ^a^	0.089
CoM(deg)							
Hip	15.92 ± 2.49	19.27 ± 1.99	18.95 ± 2.29	0.615	-	-	-
Knee	22.13 ± 1.41	23.85 ± 1.74	24.94 ± 1.46	0.503	-	-	-
Perimeter (deg)							
Stance phase	138.32 ± 3.15	125.55 ± 5.39	114.09 ± 4.40	**<0.001** ^a^	0.127	**<0.001** ^a^	0.075
Swing phase	34.40 ± 2.65	21.08 ± 2.32	23.45 ± 2.40	**0.002** ^a^	**0.001** ^a^	**0.006** ^a^	0.447
Total	172.73 ± 4.75	146.63 ± 7.24	136.58 ± 5.80	**0.001** ^a^	**0.009** ^a^	**<0.001** ^a^	0.246
Area (deg^2^)							
Stance phase	550.93 ± 28.23	402.76 ± 38.40	398.02 ± 28.24	**0.002** ^a^	**0.006** ^a^	**<0.001** ^a^	0.764
Swing phase	930.89 ± 44.86	593.51 ± 56.18	629.79 ± 51.59	**<0.001** ^a^	**<0.001** ^a^	**<0.001** ^a^	0.603
Total	1481.91 ± 63.39	996.28 ± 88.71	1023.51 ± 75.90	**<0.001** ^a^	**<0.001** ^a^	**<0.001** ^a^	0.873

Values presented as mean ± standard deviation. Bold values indicate statistically significant differences (p < 0.05).

^a^ indicates statistically significant differences.

### 3.2 Movement complexity differences based on sample entropy

The sample entropy analysis revealed significant differences in joint movement complexity among the three groups, as shown in Table 4. The hip joint sample entropy of the KOA group 0.30 ± 0.01 increased significantly by 25.0% compared to the Healthy group 0.24 ± 0.01, while the postoperative hip joint sample entropy of the TKA group 0.21 ± 0.01 decreased significantly by 30.0% compared to the KOA group, recovering to a level lower than that of the Healthy group. The ankle joint sample entropy of the KOA group 0.38 ± 0.07 increased by 35.7% compared to the Healthy group, while the TKA group showed a 21.1% decrease compared to the KOA group. The knee joint sample entropy changes exhibited a unique pattern: the KOA group showed a 20.0% increase compared to the Healthy group, but the TKA group was significantly lower than the KOA group postoperatively.

**TABLE 4:** Comparison of lower limb joint movement complexity among the three groups.

Features	Healthy	KOA	TKA	P-value	Multiple comparison		
					HvsK	HvsT	KvsT
SE_hip_	0.24 ± 0.01	0.30 ± 0.01	0.21 ± 0.01	**<0.001** ^a^	**<0.001** ^a^	0.019	**<0.001** ^a^
SE_Knee_	0.20 ± 0.01	0.24 ± 0.01	0.15 ± 0.01	**0.003** ^a^	0.275	0.059	**<0.001** ^a^
SE_ankle_	0.28 ± 0.06	0.38 ± 0.07	0.30 ± 0.07	**<0.001** ^a^	**<0.001** ^a^	0.537	**0.007** ^a^

Values presented as mean ± standard deviation. Bold values indicate statistically significant differences (p < 0.05).

^a^ indicates statistically significant differences.

### 3.3 Comparative analysis of multidimensional features

To control for the influence of confounding factors such as age, sex, and BMI, ANCOVA was employed to compare gait features between groups. The results showed that, after adjusting for covariates, multiple key features still exhibited significant intergroup differences, as presented in Table 5. The hip joint sample entropy demonstrated the largest effect size for intergroup differences, followed by total area and swing phase area, indicating that these metrics play important roles in distinguishing between groups. Knee joint RoM and ankle joint sample entropy also showed strong discriminative capabilities. In contrast, hip and knee joint CoM and hip joint RoM did not exhibit significant differences.

**TABLE 5:** ANCOVA results for gait features after adjusting for age, sex, and BMI.

Features	F-value	P-value	Partial η^2^
Total Area	14.433	**<0.001** ^a^	0.329
Swing Phase Area	14.427	**<0.001** ^a^	0.328
Stance Phase Area	8.103	**<0.001** ^a^	0.216
Total Perimeter	7.513	**0.001** ^a^	0.203
Swing Phase Perimeter	6.670	**0.002** ^a^	0.184
Stance Phase Perimeter	5.345	**0.007** ^a^	0.153
Hip CoM	0.009	0.991	0.000
Knee CoM	0.370	0.692	0.012
Hip RoM	2.414	0.098	0.076
Knee RoM	12.914	**<0.001** ^a^	0.305
SE Hip	26.267	**<0.001** ^a^	0.471
SE Knee	3.489	**0.037** ^a^	0.106
SE Ankle	11.952	**<0.001** ^a^	0.288

^a^ indicates statistically significant differences Bold values indicate statistically significant differences (p < 0.05).

To control for the effects of population heterogeneity such as the affected side in KOA and the surgical side in TKA, ANCOVA was employed to compare gait features between groups. Multiple key features still exhibited significant intergroup differences, as shown in Table 6. The hip joint sample entropy demonstrated the largest effect size for intergroup differences, followed by stance phase perimeter and ankle joint sample entropy. Hip joint RoM, knee joint RoM, and total perimeter also showed strong discriminative capabilities, with effect sizes of 0.762, 0.649, and 0.710, respectively. Although knee joint CoM, hip joint CoM, and swing phase area also presented significant differences, their effect sizes were relatively small, while swing phase perimeter did not show significant differences.

**TABLE 6:** ANCOVA results for gait features after controlling for side differences.

Features	F-value	P-value	Partial η^2^
Total Area	7.340	**0.002** ^a^	0.284
Swing Phase Area	4.741	**0.014** ^a^	0.204
Stance Phase Area	9.353	**<0.001** ^a^	0.335
Total Perimeter	45.385	**<0.001** ^a^	0.710
Swing Phase Perimeter	0.801	0.456	0.041
Stance Phase Perimeter	73.066	**<0.001** ^a^	0.798
Hip CoM	8.255	**0.001** ^a^	0.300
Knee CoM	21.344	**<0.001** ^a^	0.535
Hip RoM	59.305	**<0.001** ^a^	0.762
Knee RoM	34.240	**<0.001** ^a^	0.649
SE Hip	148.995	**<0.001** ^a^	0.889
SE Knee	16.121	**<0.001** ^a^	0.465
SE Ankle	49.915	**<0.001** ^a^	0.729

^a^ indicates statistically significant differences. Bold values indicate statistically significant differences (p < 0.05).

**FIGURE 5:**
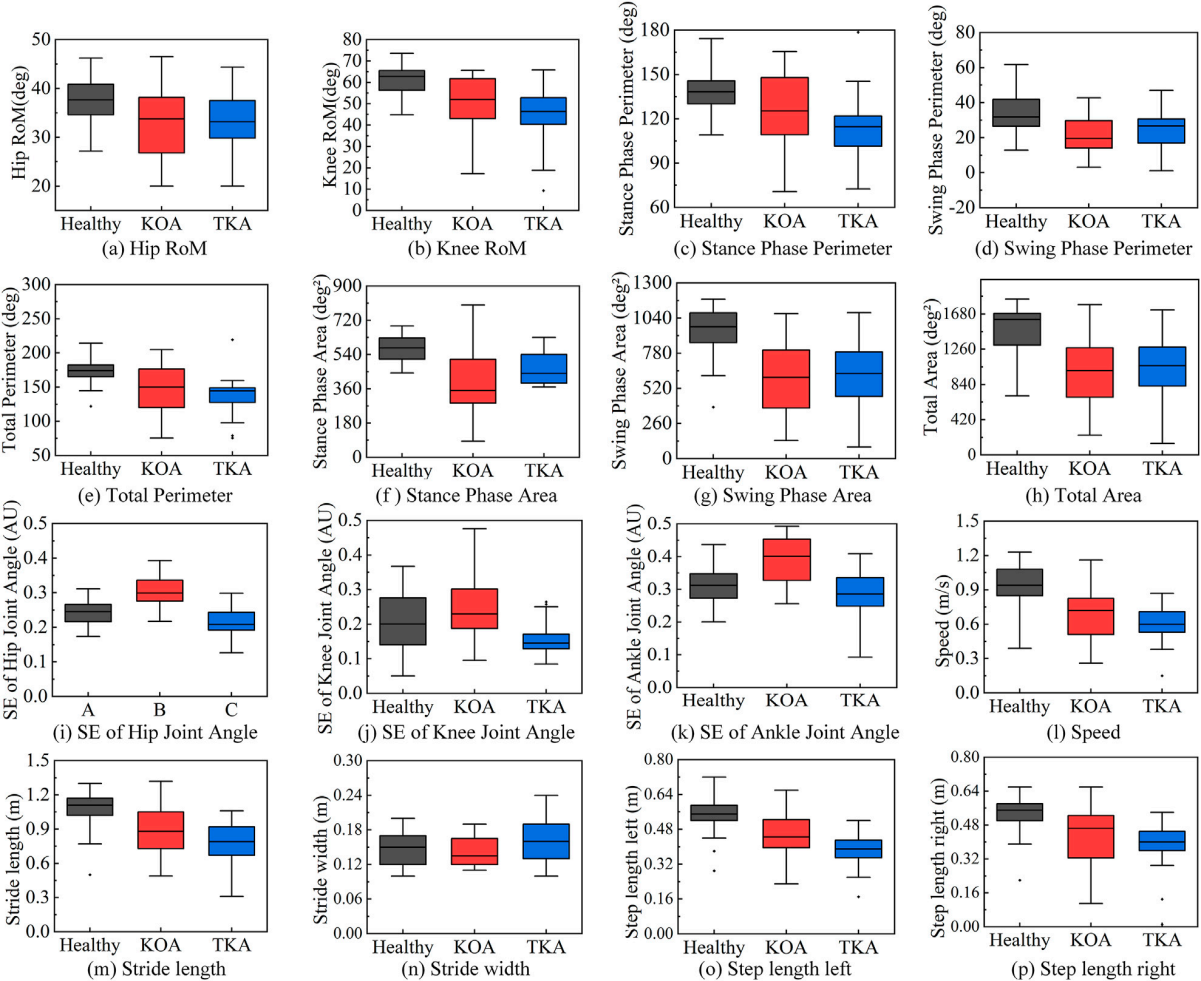
Comparative analysis of multidimensional gait features. **(a)** Hip RoM. **(b)** Knee RoM. **(c)** Stance phase perimeter. **(d)** Swing phase perimeter. **(e)** Total perimeter. **(f)** Stance phase area. **(g)** Swing phase area. **(h)** Total area. **(i)** SE of hip joint angle. **(j)** SE of knee joint angle. **(k)** SE of ankle joint angle. **(l)** Speed. **(m)** Sride length. **(n)** Stride wifth. **(o)** Step length left. **(p)** Step length right.

The comparative analysis of multidimensional gait features is shown in Figure 5, revealing the significant advantages of the multidimensional evaluation system over traditional spatiotemporal parameters. Cyclogram analysis demonstrated that the hip joint (a) and knee joint (b) RoM in the Healthy group were significantly greater than those in the KOA and TKA groups. The swing phase perimeter (d) and area (g), stance phase perimeter (c) and area (f), as well as total perimeter (e) and area (h) in the Healthy group were significantly larger than those in the KOA and TKA groups. In terms of stance phase area, the TKA group also showed significant improvement compared to the KOA group. The complexity of hip (i), knee (j), and ankle (k) joint angle variations in the KOA group was significantly higher than that in both the Healthy and TKA groups. Among the spatiotemporal parameters across the three groups, stride length (m), left step length (o), right step length (p), and gait speed (l) in the Healthy group were significantly higher than those in the KOA and TKA groups, while step width (n) showed no significant differences among the Healthy, KOA, and TKA groups.

**FIGURE 6:**
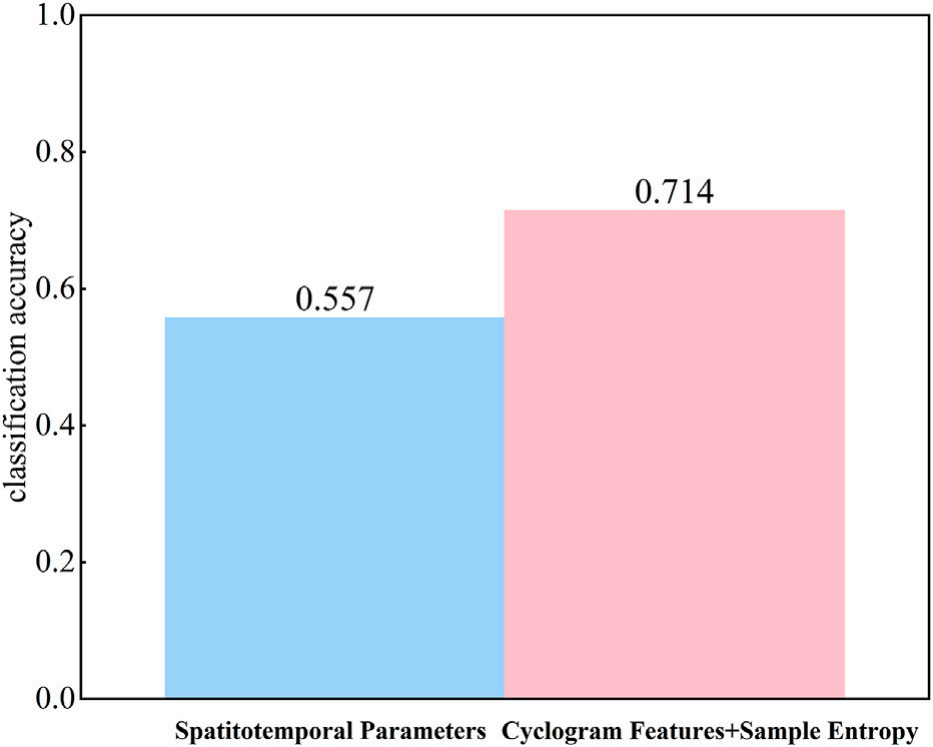
Comparison of LDA classification accuracy.

To further directly validate the superiority of multidimensional feature fusion compared to traditional spatiotemporal parameters, linear discriminant analysis (LDA) was employed to evaluate the overall discriminative ability of the two feature sets, with the average accuracy calculated using 5-fold cross-validation, as shown in Figure 6. The LDA results demonstrated that the classification accuracy based on traditional spatiotemporal parameters was 55.7%, while the classification accuracy based on multidimensional feature fusion reached 71.4%.

### 3.4 Classification performance of feature fusion

The classification performance of the four machine learning models in gait analysis is shown in Table 7, where the multidimensional feature combination demonstrated the best performance across all models. Among them, RF achieved the most outstanding performance with an accuracy of 96.93%, significantly superior to its performance with single-feature inputs. DT achieved an accuracy of 92.44% under multidimensional features, representing an improvement of 6.43–19.28 percentage points compared to single-feature inputs, further validating the importance of multiparameter evaluation. SVM and KNN also achieved accuracies of 90.29% and 88.98%, respectively, after feature fusion, indicating the universal advantages of the multidimensional feature analysis strategy.

**TABLE 7:** Comparison of classification performance of four machine learning.

Model	Feature selection	Acc	Pre	Recall	F1 score
RF	Cyclogram Features + Sample Entropy	**96.93%**	**97.17%**	**97.52%**	**97.32%**
	Cyclogram Features	89.73%	86.01%	87.66%	87.51%
	Sample Entropy	75.66%	76.51%	75.50%	74.95%
	Spatiotemporal Parameters	83.00%	83.50%	82.50%	83.00%
DT	Cyclogram Features + Sample Entropy	**92.44%**	**92.63%**	**93.37%**	**93.54%**
	Cyclogram Features	86.01%	87.66%	87.51%	86.54%
	Sample Entropy	73.16%	73.40%	73.31%	73.40%
	Spatiotemporal Parameters	82.00%	82.50%	81.50%	82.00%
SVM	Cyclogram Features + Sample Entropy	**90.29%**	**92.63%**	**93.37%**	**93.54%**
	Cyclogram Features	78.38%	78.91%	78.31%	79.80%
	Sample Entropy	78.66%	77.06%	77.21%	78.77%
	Spatiotemporal Parameters	81.00%	81.50%	80.50%	81.00%
KNN	Cyclogram Features + Sample Entropy	**88.98%**	**88.66%**	**88.74%**	**88.94%**
	Cyclogram Features	74.00%	73.63%	72.45%	72.28%
	Sample Entropy	66.28%	66.54%	66.65%	66.31%
	Spatiotemporal Parameters	75.90%	76.31%	74.35%	76.05%

Bold denotes the highest score.

**FIGURE 7:**
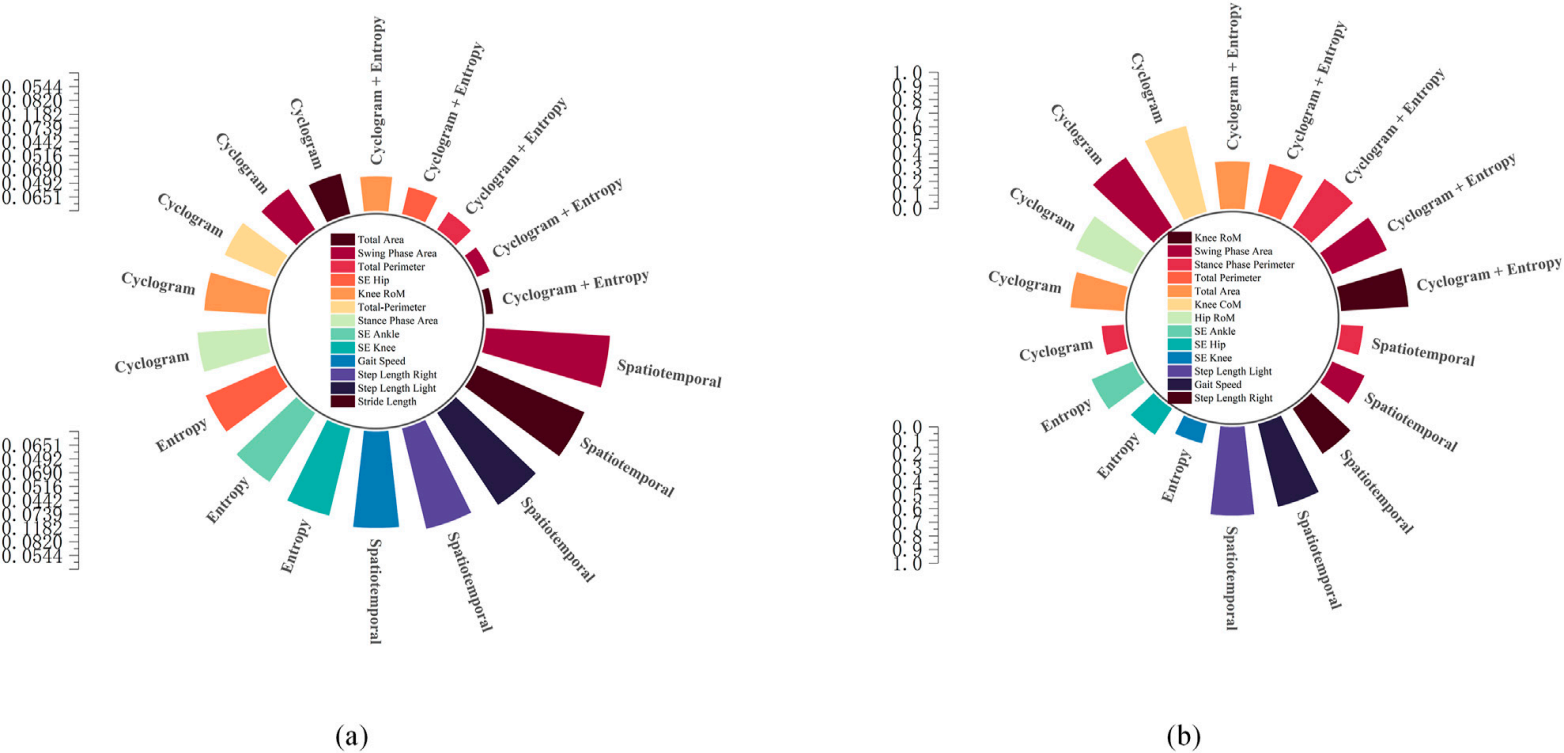
Comparison of the top five feature importances. **(a)** RF; **(b)** SVM.

A comparison of the top five feature importances across multiple models is shown in Figure 7. Under spatiotemporal parameter input: in the SVM model (b), left step length ranked as the most important feature, followed by gait speed; in the RF model (a), gait speed was the core feature. Under sample entropy input: in the SVM model, ankle joint sample entropy ranked first, with hip joint sample entropy second; in the RF model, hip joint sample entropy ranked first. Under cyclogram morphological feature input: in the SVM model, knee joint CoM and swing phase area ranked first and second, respectively, with total area ranking fourth; in the RF model, total area and swing phase area occupied the top two positions. Under multidimensional feature fusion input: in the SVM model, knee joint RoM and swing phase area dominated discrimination; in the RF model, total area and swing phase area maintained their core positions.

**FIGURE 8:**
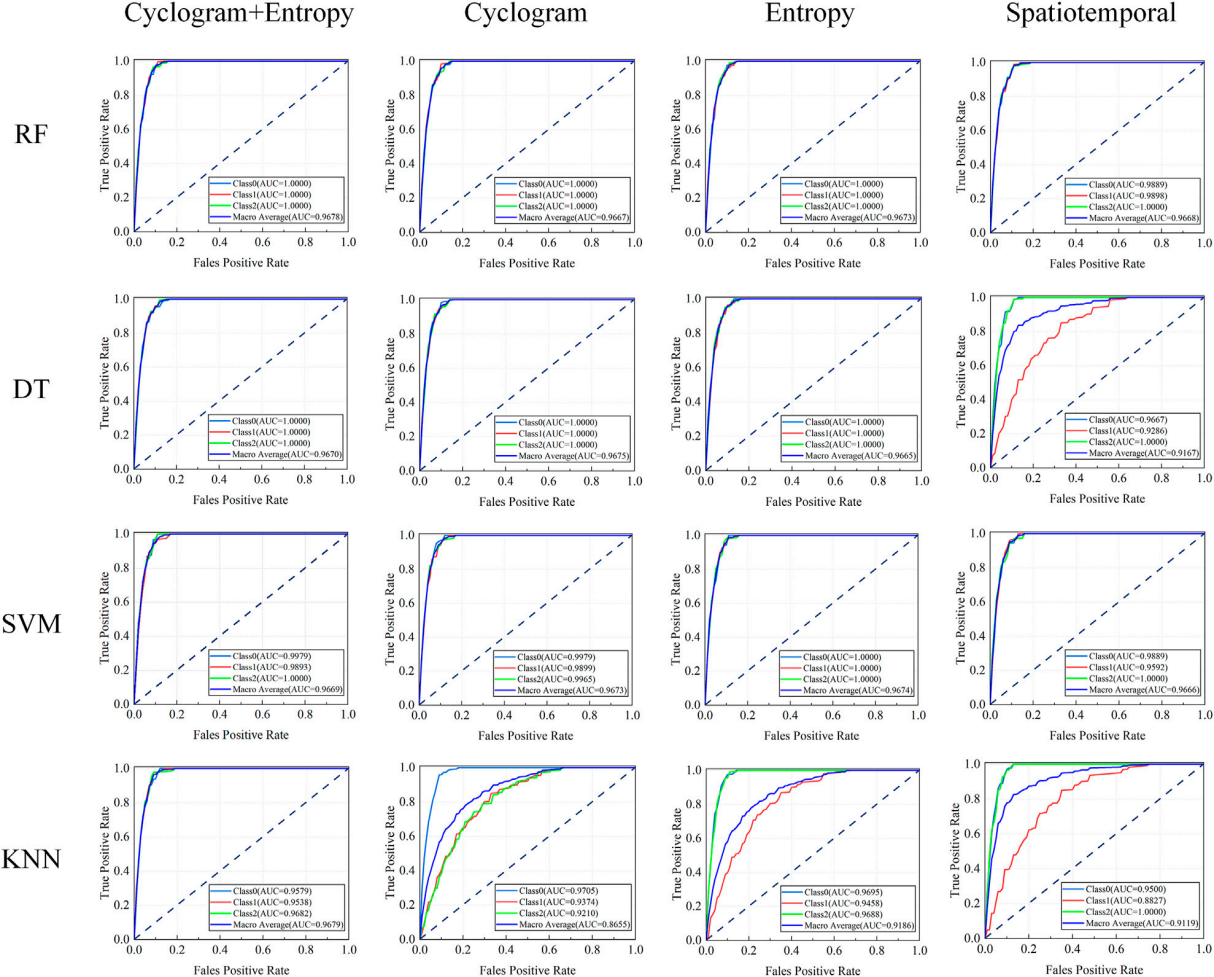
ROC curves of RF/SVM/DT/KNN models.

The Bootstrap method was used to compare the AUC differences among the four types of models, as shown in Figure 8. Compared to single spatiotemporal parameter input, the performance of all models showed systematic improvement after integrating multidimensional features, a trend particularly evident in the area under the ROC curve metric. The RF model achieved a macro-average AUC of 0.9678 under fused features, representing a significant improvement over single-feature inputs; the SVM macro-average AUC reached 0.9670, DT reached 0.9669, and KNN reached 0.9679, indicating that feature fusion effectively enhanced the models’ ability to capture complex patterns and significantly optimized classification boundary determination accuracy (Ren et al., 2023).

## 4 Discussion

This study achieved synchronous quantitative assessment of gait dynamic coordination and movement complexity in patients with KOA and post-TKA by integrating hip-knee cyclogram morphological features and joint angle sample entropy. Multidimensional feature analysis revealed a multidimensional dissociation phenomenon characterized by “decreased cyclogram features with increased sample entropy” in the KOA group. The Healthy group exhibited high cyclogram features and low sample entropy characteristics, which aligns with van den Noort et al. (2022) reporting that muscle atrophy in KOA patients primarily involves the vastus medialis, whose atrophy leads to weakened knee extension strength, and Yamauchi et al. (2020) observing that muscle atrophy in KOA patients mainly affects the quadriceps and biceps femoris, potentially caused by joint pain, inflammatory responses, impaired neural activation, and reduced activity. Such muscular atrophy results in decreased hip and knee extension/flexion strength, consequently affecting gait complexity and coordination. In contrast, the TKA group displayed unique postoperative characteristics of improved stance phase area and low sample entropy, indicating that TKA improves knee function through precise prosthetic alignment and size adjustment. To control for the influence of confounding factors such as age, sex, and BMI, as well as population heterogeneity arising from the affected side in KOA and the surgical side in TKA, ANCOVA was employed. The results demonstrated that these factors did not significantly affect the feature patterns.

Comparison with traditional spatiotemporal parameters validated the superiority of multidimensional features. Although spatiotemporal parameters can distinguish between the healthy group and the KOA group (Kösesoy, 2023), they fail to effectively differentiate between the KOA group and the TKA group. In contrast, through multidimensional gait feature comparative analysis, the differences between the TKA group and the KOA group become clearly apparent. Feature importance analysis provides interpretable evidence for the conclusions. The RF model revealed that total cyclogram area and swing phase area are core discriminative features, confirming the indicative role of joint dynamic coordination in pathological gait. This finding aligns with the method employed by Pau et al. (2022b) in their study on multiple sclerosis patients, which used hip-knee cyclograms to quantify coordination; their research similarly demonstrated high sensitivity of cyclogram area and perimeter to disability levels. The synergistic contribution of ankle joint sample entropy and knee joint RoM highlights the value of assessing multi-joint movement complexity, consistent with the view proposed by Zanin et al. (2022) that “entropy increase reflects diminished motor control.” In the SVM model, the combination of knee joint RoM and swing phase area dominated classification decisions, further illustrating that the interaction between coordination impairment and complexity compensation is key to distinguishing the three groups.

Although compared to the information set-based decision tree (IFS-DT) method proposed by Balakrishnan et al. (2024), this study not only achieved high-precision classification but also integrated cyclogram morphological features and sample entropy in a multidimensional manner, enabling a more comprehensive capture of joint movement coordination and complexity characteristics, thereby providing richer quantitative indicators for gait assessment; compared to the stroke assessment method based on multidimensional gait parameters by Wang et al. (2025), although its spatiotemporal parameters were effective for stroke grading, its recognition accuracy for mild cases was only 58.33%–66.67%, still limited by the unidimensional nature of the features. This study further integrated the clinical translation pathway into the supplementary materials, constructing an intelligent assessment closed loop encompassing data acquisition, feature extraction, model inference, and clinical interpretation, laying the technical foundation for translation into real-world clinical scenarios. This framework not only provides dynamic, quantitative, and interpretable assessment metrics for post-TKA rehabilitation but also holds the potential to reshape decision-making patterns in orthopedic rehabilitation. By continuously monitoring the recovery trajectory of coordination metrics and the normalization process of complexity parameters, it offers evidence-based support for rehabilitation phase determination, individualized plan adjustment, and prognosis judgment, thereby promoting a shift in rehabilitation medicine from an experience-oriented to a data-driven paradigm. Future work will enhance model generalization through multicenter clinical validation and explore integration with wearable sensing, IoT, and digital twin technologies to build an intelligent rehabilitation management ecosystem covering hospital-community-home settings, ultimately achieving the widespread application of precision rehabilitation medicine.

## 5 Limitations

Although the multidimensional gait feature fusion algorithm proposed in this study demonstrates strong discriminative ability in distinguishing healthy individuals, KOA patients, and post-TKA populations, and offers a novel analytical perspective, several noteworthy limitations remain. This study adopted a cross-sectional design and did not include preoperative baseline data or longitudinal follow-up at multiple postoperative time points for TKA patients, making it difficult to determine to what extent the observed gait changes can be attributed to surgical recovery effects, adaptive compensatory strategies, or residual preoperative patterns, thereby limiting causal inferences regarding intervention effectiveness. Additionally, the sample size was relatively limited, particularly in subgroup analyses such as different surgical types or rehabilitation stages, where imbalanced distributions may affect the robustness and generalization ability of the machine learning models. Although statistical control was applied for variables such as age, sex, and BMI using analysis of covariance, and heterogeneity due to affected side and surgical side was considered, other unmeasured confounding factors—such as muscle strength levels, pain perception, and joint stability—may still have potential influences on the results. Finally, as all participants were recruited from a single center and were not stratified by key rehabilitation windows, the generalizability of the conclusions requires further validation through larger-scale, multicenter, prospective longitudinal studies.

## 6 Conclusion

This study established a multidimensional gait feature fusion algorithm that integrates hip-knee cyclogram morphological features and joint angle sample entropy, effectively distinguishing gait differences among healthy individuals, KOA patients, and post-TKA patients. It reveals the movement compensation mechanisms in KOA patients and the partial recovery of motor function after TKA, validated by machine learning models, with classification performance superior to traditional single-dimensional methods. The feature extraction approach based on cyclogram morphology and sample entropy captures subtle movement control differences that cannot be reflected by traditional spatiotemporal parameters, providing an interpretable quantitative tool for analyzing KOA pathological mechanisms and assessing post-TKA rehabilitation.

## Data Availability

The raw data supporting the conclusions of this article will be made available by the authors, without undue reservation.
