# Supplementary material for: Application of multidimensional gait feature fusion algorithm in gait assessment for patients with knee osteoarthritis
Source: Front Bioeng Biotechnol. 2025 Sep 19;13:1645162. doi: 10.3389/fbioe.2025.1645162 (PMC12491203; doi:10.3389/fbioe.2025.1645162)
Supplement: Supplementary file 1 [file Supplementaryfile1.docx]

Description of Supplementary Materials

1. **Example of Machine Learning Input Data Structure**

An example of the input data structure for multidimensional feature fusion is shown in Table 1. This table presents the complete input format for the machine learning model, including each subject's unique ID, morphological features extracted from hip-knee cyclograms, joint CoM features, joint RoM features, sample entropy features, and the final group classification label (Healthy/KOA/TKA). In this structured data format, each row represents a complete feature vector for one subject. For example, Subject ID 1 (Healthy) has feature values denoted by "XX" representing actual measured values. The last column, "Group," serves as the classification target for supervised learning, enabling machine learning algorithms to learn the mapping between features and clinical groups, thereby achieving automatic discrimination among the three populations. This tabular design integrates multidimensional information including dynamic coordination, movement complexity, and traditional parameters, providing standardized input for classification models such as Random Forest (RF). The area parameters are measured in degrees squared (deg²), perimeter and range of motion (RoM) in degrees (deg), and sample entropy is a dimensionless complexity metric. Together, all features form a 13-dimensional feature space.

Table 1 Input for fused multidimensional features

| ID | Swing phase area | Stance phase area | Total area | Swing phase perimeter | Stance phase perimeter | Total perimeter | Hip CoM | Knee CoM | Hip RoM | Knee RoM | SE hip | SE knee | SE ankle | Group |
| --- | --- | --- | --- | --- | --- | --- | --- | --- | --- | --- | --- | --- | --- | --- |
| 1 | XX | XX | XX | XX | XX | XX | XX | XX | XX | XX | XX | XX | XX | Healthy |

The input structure for cyclogram morphological features is shown in Table 2. This table details the input format specifically designed for hip-knee dynamic coordination in the machine learning model, including each subject's unique ID and the complete set of morphological parameters extracted from the hip-knee cyclogram: Swing phase area, Stance phase area, Total area, Swing phase perimeter, Stance phase perimeter, Total perimeter, Hip CoM, Knee CoM, Hip RoM, and Knee RoM, with the final group classification label (Healthy/KOA/TKA) as the supervised target. In this structured data format, each row corresponds to a subject's coordination feature vector. For example, Subject ID 1 (Healthy) has feature values "XX" representing actual measured joint motion coordination parameters. Here, area parameters quantify the spatial coverage of the cyclogram, perimeter parameters reflect the trajectory length of joint angles, centroid coordinates characterize the average angular position of joints, and range of motion (RoM) indicators describe the extreme range of joint motion. Together, these parameters form a 10-dimensional pure morphological feature space, enabling classification models to independently learn the mapping relationship between cyclogram features and clinical groups.

Table 2 Input of cyclogram morphological features

| ID | Swing phase area | Stance phase area | Total area | Swing phase perimeter | Stance phase perimeter | Total perimeter | Hip CoM | Knee CoM | Hip RoM | Knee RoM | Group |
| --- | --- | --- | --- | --- | --- | --- | --- | --- | --- | --- | --- |
| 1 | XX | XX | XX | XX | XX | XX | XX | XX | XX | XX | Healthy |

The input structure for sample entropy features is shown in Table 3. This table presents the streamlined input format specifically designed to quantify joint movement complexity in the machine learning model, including each subject's unique ID and the sample entropy measurements for the hip, knee, and ankle joints: SE hip, SE knee, and SE ankle, with the final group classification label (Healthy/KOA/TKA) as the supervised target. In this compact data structure, each row corresponds to a subject's movement complexity feature vector. For example, Subject ID 1 (Healthy) has values "XX" representing the actual calculated sample entropy values. These dimensionless scalars characterize the complexity of gait movement by quantifying the regularity of joint angle time series. The three-dimensional entropy values collectively form a 3-dimensional complexity feature space, enabling classification models to independently learn the mapping relationship between movement complexity and clinical groups.

Table 3 Input of sample entropy features

| ID | SE hip | SE knee | SE ankle | Group |
| --- | --- | --- | --- | --- |
| 1 | XX | XX | XX | Healthy |

The input structure for spatiotemporal parameters is shown in Table 4. This table presents the input format of traditional gait spatiotemporal parameters for the machine learning model, including each subject's unique ID and five basic spatiotemporal metrics: Step Length right, Step Length left, Step width, Gait speed, and Stride length, with the final group classification label (Healthy/KOA/TKA) as the supervised target. In this standardized data structure, each row corresponds to a subject's spatiotemporal feature vector. For example, Subject ID 1 (Healthy) has values "XX" representing actual measured kinematic parameters. Here, left and right step lengths quantify lower limb asymmetry, step width reflects dynamic balance ability, gait speed comprehensively characterizes mobility efficiency, and stride length indicates overall movement amplitude. These conventional parameters collectively form a 5-dimensional spatiotemporal feature space, enabling classification models to learn the mapping relationship between traditional gait parameters and clinical groups.

Table 4 Input of spatiotemporal parameters

| ID | Step Length right | Step Length light | Step width | Gait speed | Stride length | Group |
| --- | --- | --- | --- | --- | --- | --- |
| 1 | XX | XX | XX | XX | XX | Healthy |

1. **Feature Selection Strategy**

The comparison of model performance using different feature selection methods on multidimensional feature fusion is shown in Table 5. All combinations of models and feature selection methods were tested across four feature sets, with AUC serving as the core performance metric. The table below extracts and summarizes the performance of different feature selection methods on the most important multidimensional feature fusion configuration. On the most discriminative multidimensional feature fusion, the ANOVA method achieved the best overall performance. Among the four machine learning models, ANOVA yielded the highest AUC values for three models: RF, SVM, and KNN. The RFE method performed slightly better than ANOVA only on the DT model, demonstrating its effectiveness in specific model contexts. The LASSO method showed relatively weaker overall performance in this study. The final selection of the ANOVA test as the core feature selection strategy was based on its optimal balance among performance, efficiency, and interpretability. First, ANOVA achieved the most robust average classification performance on the most discriminative feature combination, meeting the core requirement of model generalizability. Second, as a filter method, its computational efficiency is significantly higher than that of RFE and LASSO, which require iterative retraining—this is crucial for processing high-dimensional biomechanical data. Third, ANOVA selects features based on their statistical differences across groups, resulting in features with clear biomechanical significance. This enhances the interpretability of the research findings, making them easier to understand and accept. Additionally, compared to more complex embedded methods, ANOVA's selection process is independent of the classification model, reducing the risk of overfitting on limited sample sizes to some extent.

Table 5 Comparison of model performance using different feature selection methods on fused multidimensional features

| Model | ANOVA (AUC) | RFE (AUC) | LASSO (AUC) |
| --- | --- | --- | --- |
| RF | **0.9633** | 0.9600 | 0.9400 |
| DT | 0.9331 | **0.9558** | 0.9400 |
| SVM | **0.9348** | 0.9259 | 0.9223 |
| KNN | **0.9350** | 0.9146 | 0.8931 |

1. **Cross-validation of sample entropy**

To ensure the reliability of sample entropy as an evaluation metric for movement complexity, this study conducted a systematic cross-validation analysis by comparing its correlations with multiple time-domain and frequency-domain features. As shown in Table 6, the time-domain features primarily include the coefficient of variation (CV), while the frequency-domain features encompass several metrics such as mean frequency, spectral entropy, total power, and dominant frequency power. The analysis results reveal distinct group- and joint-specific relationships between sample entropy and different features: in the KOA group, the ankle and hip joints exhibited the highest sample entropy values, accompanied by higher movement variability and relatively lower total power values, suggesting that the movement patterns of KOA patients may demonstrate higher complexity and instability; in contrast, the TKA group showed the lowest sample entropy values and dominant frequency power in the knee joint but the highest mean frequency and spectral entropy, indicating that postoperative patients may adopt a more rhythmic yet energetically dispersed movement strategy; the Healthy group displayed intermediate characteristics, with the lowest sample entropy values and the highest total power in the knee joint, reflecting an efficient and stable gait pattern.

Table 6 Comparison of joint motion feature parameters among the three groups of subjects

| Group | Joint | CV | Frequency | Spectral Entropy | Sample Entropy | Total Power | Dominant Frequency Power |
| --- | --- | --- | --- | --- | --- | --- | --- |
| Healthy | Ankle | 0.33±3.50 | 2.24±0.27 | 1.31±0.17 | 0.28±0.06 | 215434.8±91914.7 | 104377.2±49135.5 |
| Healthy | Hip | 2.23±6.53 | 1.15±0.07 | 0.33±0.11 | 0.24±0.01 | 752232.7±231100.0 | 700576.1±222412.9 |
| Healthy | Knee | 0.96±0.37 | 2.79±0.27 | 1.44±0.14 | 0.20±0.01 | 1172730±320354.3 | 629232.7±195810.6 |
| KOA | Ankle | 2.30±6.43 | 2.16±0.50 | 1.26±0.30 | 0.38±0.07 | 203014.8±116615.4 | 105944.5±64007.4 |
| KOA | Hip | 0.77±0.44 | 1.18±0.12 | 0.35±0.12 | 0.30±0.01 | 571627.5±247529.8 | 529124.3±237973.4 |
| KOA | Knee | 0.76±0.54 | 2.77±0.36 | 1.44±0.18 | 0.24±0.01 | 845945.6±455032.6 | 445617.8±270659.6 |
| TKA | Ankle | 0.36±3.02 | 2.08±0.40 | 1.24±0.32 | 0.30±0.07 | 141299.9±76031.2 | 75450.16±41690.5 |
| TKA | Hip | 2.46±6.78 | 1.22±0.27 | 0.41±0.23 | 0.21±0.01 | 543126.5±245701.7 | 493084.7±232179.0 |
| TKA | Knee | 0.62±0.33 | 3.02±0.45 | 1.59±0.24 | 0.15±0.01 | 572623.3±292113.8 | 245053.6±128263.5 |

Values presented as mean ± standard deviation

The correlation heatmap analysis, as shown in Figure 1, reveals a complex network of interrelationships among various movement features. As illustrated in Figure 1(a)-(c), for the hip, knee, and ankle joints in the Healthy group, the mean frequency and spectral entropy demonstrate high consistency, while total power and dominant frequency power also exhibit a strong positive correlation. In the KOA group, Figures 1(d)-(f) show characteristic alterations in correlations for the hip, knee, and ankle joints, where the relationship between sample entropy and frequency features weakens or even becomes negative. In contrast, the TKA group, depicted in Figures 1(g)-(i), displays unique correlation patterns across joints: the knee joint shows a positive correlation between sample entropy and both total power and dominant frequency power, while this relationship is significantly weakened in the ankle joint. These group- and joint-specific correlation patterns provide important visual evidence for understanding adaptive changes in motor control mechanisms under different pathological conditions. However, future studies incorporating direct measurements of neural activity, such as electromyography, are needed to elucidate the physiological basis of these correlation patterns.

Figure 1 Correlation Heatmap

1. **Clinical Application of the Model**

The multidimensional gait feature fusion algorithm developed in this study aims to be translated into an objective quantitative tool for clinical practice, providing data-driven support for postoperative rehabilitation monitoring after TKA. Figure 2 clearly illustrates the complete workflow of the model, from data acquisition to clinical decision support. The process begins with the collection of raw kinematic data during patient walking using a three-dimensional motion capture system in a standardized walkway laboratory. Subsequently, the system automatically preprocesses the data and extracts two core types of features: first, hip-knee cyclogram morphological features to quantify dynamic coordination between lower limb joints, and second, sample entropy of hip, knee, and ankle joint angles to quantify the complexity of joint movements. These features are combined and input into a pre-trained optimal machine learning model for real-time analysis. The model's output is not a simple "black-box" label but includes rich interpretable information: first, the classification result, which provides the probability that the subject's gait pattern belongs to the healthy, KOA, or post-TKA category, offering an objective reference for clinical diagnosis; second, a feature contribution analysis, which identifies the specific coordination or complexity metrics that led to the classification result, directly revealing the patient's abnormal compensatory patterns.

The clinical significance of this workflow lies in its successful integration of a high-precision algorithm, validated in a research environment, into a framework aligned with clinical practice logic, addressing current pain points in clinical assessment. The model's value is particularly prominent for postoperative TKA rehabilitation management. Physicians can periodically assess patients and, by comparing results across evaluations, quantitatively track recovery rates of coordination metrics and reduction magnitudes of complexity metrics. This enables objective evaluation of rehabilitation effectiveness, identification of plateaus in the recovery process, and timely adjustment of rehabilitation strategies, achieving truly personalized and refined rehabilitation management. In summary, this algorithm transforms complex gait data analysis into intuitive, quantitative clinical reports, significantly enhancing the ability of physicians and rehabilitation therapists to understand and address gait disorders. It holds promise as a novel and powerful digital clinical auxiliary tool.

Figure 2 Workflow chart of rehabilitation monitoring based on a multidimensional gait feature fusion algorithm
